# 靶向二代测序技术在血液病患者社区获得性呼吸道病毒感染中的诊断价值

**DOI:** 10.3760/cma.j.cn121090-20241224-00586

**Published:** 2025-07

**Authors:** 雪宜 罗, 雨辰 姚, 瑞 马, 慧芳 王, 露 柏, 伟 韩, 翼飞 程, 菲菲 唐, 晓军 黄, 于谦 孙

**Affiliations:** 北京大学人民医院、北京大学血液病研究所、国家血液系统疾病临床医学研究中心，北京 100044 Peking University People's Hospital, Peking University Institute of Hematology, National Clinical Research Center for Hematologic Disease, Beijing 100044, China

**Keywords:** 社区获得性呼吸道病毒, 咽拭子, 靶向二代测序, 血液病, Community-acquired respiratory virus, Pharyngeal swab, Targeted next-generation sequencing, Hematological diseases

## Abstract

**目的:**

探讨血液病患者咽拭子靶向二代测序（Targeted next-generation sequencing，tNGS）在检测社区获得性呼吸道病毒（Community-acquired respiratory viruses，CARV）中的诊断价值。

**方法:**

回顾性分析2023年9月至2024年4月期间，在北京大学人民医院血液科因出现可疑感染表现同时接受咽拭子tNGS及CARV核酸扩增（PCR）检测的64例血液病患者临床数据及检验结果，评估tNGS与CARV PCR结果的一致性及其诊断CARV的效力。

**结果:**

64例患者中，临床诊断呼吸道感染患者29例（1例巨细胞病毒肺炎，其余均CARV阳性），其他原因引起发热/呼吸道改变患者35例（14例为肺外部位感染，21例考虑非感染因素所致）。中位随访215.5（7，271）d，PCR方法共检出CARV 7种24株，tNGS方法检出CARV 8种25株，以PCR CARV为标准，tNGS CARV的灵敏度为85.0％，特异度88.6％，阳性预测值为77.3％，阴性预测值为92.9％，准确率为87.5％，两种检测方法的一致性良好，Kappa值为0.717（*P*<0.001）。

**结论:**

咽拭子tNGS或可作为PCR检测的替代方案诊断血液病患者CARV感染。

## Abstract (en)

**Objective:**

To evaluate the diagnostic value of targeted next-generation sequencing（tNGS）of throat swab samples for detecting community-acquired respiratory viruses（CARV）in patients with hematological diseases.

**Methods:**

Clinical and laboratory data from 64 episodes involving patients with hematological diseases and suspected infections—who underwent both pharyngeal swab tNGS and CARV polymerase chain reaction（PCR）testing concurrently—were retrospectively analyzed. The cases were drawn from the Department of Hematology, Peking University People's Hospital, between September 2023 and April 2024. Concordance between tNGS and CARV PCR results, as well as the diagnostic performance of tNGS in detecting CARV, were evaluated.

**Results:**

Among the 64 episodes, 29 were clinically diagnosed with respiratory tract infections, including one case of cytomegalovirus pneumonia and 28 CARV-positive cases. The remaining 35 episodes involved patients with fever or respiratory symptoms attributed to other causes, including 14 with extrapulmonary infections and 21 with noninfectious etiologies. The median follow-up duration was 215.5 days（range: 7–271 days）. PCR detected 24 strains of seven CARV types, whereas tNGS detected 25 strains of eight CARV types. Using PCR results as the reference standard, the sensitivity, specificity, positive predictive value, negative predictive value and accuracy of tNGS were 85.0％, 88.6％, 77.3％, 92.9％, and 87.5％, respectively. The two methods showed good concordance（Kappa＝0.717, *P*<0.001）.

**Conclusion:**

Pharyngeal swab tNGS may serve as a viable alternative to PCR for diagnosing CARV infections in patients with hematological diseases.

血液病患者常伴有严重免疫功能缺陷，是社区获得性呼吸道病毒（Community-acquired respiratory viruses, CARV）感染的高危人群。常见的CARV包括流感病毒、呼吸道合胞病毒、冠状病毒、肠道病毒/鼻病毒、人类偏肺病毒、人类副流感病毒、人类博卡病毒和腺病毒等[1]–[2]。与非免疫抑制人群相比，血液病患者的CARV感染发生率高、病毒脱落时间长、病情进展迅速、致死率高[1],[3]–[7]。

病毒的实验室检测手段包括PCR、直接抗原检测（Direct antigen detection, DAD）、病毒分离（Virus isolation by cell culture, VIC）[1]。VIC特异度高，但需要专门的病毒学实验室，灵敏度不如PCR，检测时间相对较长；DAD具有良好的临床特异度，检测时间短，但与VIC和PCR相比灵敏度低[8]–[9]。PCR是目前CARV检测的标准方法[10]，灵敏度高、检测速度快、可量化病毒载量[11]–[15]。但以上方法都仅限于检测具有已知基因序列的病毒，而无法检测到未知序列的其他病原，因此在疑难感染、新发突发传染病等场景中应用有限。二代测序（Next-generation sequencing, NGS）技术能够无差别检测所有微生物，病原检出率明显高于包括PCR在内的常规方法（Conventional microbiological tests, CMT）[16]–[18]。目前临床常见的NGS技术主要有宏基因组测序（Metagenomic next-generation sequencing, mNGS）和靶向二代测序（Targeted next-generation sequencing, tNGS）。相比于mNGS，tNGS检测速度更快；针对多种部位样本的多项研究认为，二者诊断性能相当[16],[19]–[20]，因此tNGS具有潜在的临床应用价值。但NGS技术仍存在对阳性结果暂无统一的判读标准、与CMT结果一致性不佳、临床指导治疗意义并不明确等问题[16],[18],[21]–[22]；既往tNGS相关研究多以无菌部位标本为主，如肺泡灌洗液、血浆、骨髓、脑脊液等[20],[23]，在血液病呼吸道疾病诊治过程中存在标本不易取得、不能重复获取、阳性率低等缺陷。咽拭子是最容易收集的呼吸道病原体检测的标本类型，本研究在对临床怀疑感染的血液病患者进行咽拭子呼吸道病原PCR基础上，增加咽拭子tNGS检测，探讨咽拭子tNGS作为初步检测措施，在出现感染表现的血液病患者中的诊断价值。

## 病例与方法

一、病例资料

回顾性分析2023年9月至2024年4月在北京大学人民医院血液科因感染接受咽拭子tNGS及呼吸道病毒PCR检测的64例血液病患者，在同一天送检咽拭子tNGS及病原PCR。

二、PCR方法及结果判定

常规咽拭子采样送检，采用13种呼吸道病原体多重检测试剂盒，在一个扩增管中进行一步法RT-PCR扩增；通过毛细电泳分离不同长度的扩增产物，进而获取病原体的检测结果。

三、tNGS方法及结果判定

采用多重PCR联合NGS技术，以153种呼吸道病原的高度保守区域为靶标，设计特异性引物，在一个扩增管中进行PCR扩增，富集目标病原。然后，通过第二轮PCR连接上区分样本来源的测序接头，采用KMMinisegDx-CN基因测序仪（广州市金圻睿生物科技有限责任公司，注册证号：国械注准20203220340）进行高通量测序，获取测序数据。使用生物信息学软件，先对测序数据进行过滤，并与参考基因组进行比对，以判读病原体的检测结果。通过对样品中的人DNA进行检测，以监控样品质量。

四、定义

发热：体温高于37.3°C。低氧：血气分析氧合指数（PaO_2_/FiO_2_）小于300 mmHg（1 mmHg＝0.133 kPa）。胸部CT改变：出现新发病灶或原有病灶加重。呼吸道感染诊断标准参考第四届欧洲白血病感染大会（ECIL-4）制定指南。上呼吸道感染性疾病（Upper respiratory tract infectionsdisease, URTID）定义为在上呼吸道标本中检出CARV，同时伴有症状或体征，并排除其他病因；下呼吸道感染性疾病（Lower respiratory tract infectionsdisease, LRTID）定义为出现病理性排痰、低氧血症或肺部浸润性病变，同时呼吸道分泌物中检出CARV（优先采用感染部位采集的样本）。本文定义呼吸道CARV感染临床诊断标准：新发症状和（或）体征包括咳嗽、咽痛、气促、鼻炎、低氧、咳痰、低氧、肺部浸润，且临床医师判定与感染有关；实验室诊断标准：通过VIC、DAD、PCR或tNGS检测样本中的CARV，同时除外其他病原感染为主要致病原因的情况。移植后早期：移植后≤100 d；移植后晚期：移植后>100 d。

五、统计学处理

使用SPSS 26.0进行统计学分析。Kolmogorov-Smironv法对数据进行正态性检验。连续资料组间比较采用Student's *t*检验或Mann-Whitney *U*检验，计数资料采用卡方检验或Fisher精确概率法。计算PCR与tNGS检测CARV的灵敏度、特异度、阳性预测值、阴性预测值及准确率。采用Kappa值来量化评估诊断结果的一致性，*P*<0.05为差异有统计学意义。

## 结果

一、患者基本特征

64例出现感染表现的血液病患者中，男42例，女22例，中位年龄38.5（3~69）岁。急性髓系白血病29例，急性淋巴细胞白血病14例，骨髓增生异常综合征11例，慢性粒-单核细胞白血病5例，原发性骨髓纤维化2例，重型再生障碍性贫血2例，慢性活动性EB病毒感染1例。45例患者行造血干细胞移植，其中移植后早期14例，移植后晚期31例。临床考虑呼吸道感染患者29例（1例巨细胞病毒肺炎，其余均病程中查CARV阳性）。其他原因引起发热/呼吸道改变患者35例：21例非感染，14例其他部位感染。tNGS CARV阴性与阳性患者临床特征比较见表1，相较于tNGS CARV阴性患者，tNGS CARV阳性患者多见于移植后晚期（88.9％对55.6％，*P*＝0.023）；余临床特征两组间差异均无统计学意义。

**表1:** 64例出现感染表现的血液病患者中tNGS CARV阴性与阳性病例临床基本特征比较

临床特征	tNGS CARV阴性 （41例）	tNGS CARV阳性 （23例）	*P*值
性别（例，男/女）	30 / 11	12 / 11	0.090
年龄［岁，*M*（范围）］	41（3~69）	36（15~69）	0.966
原发病［例（％）］			0.679
ALL	11（26.8）	3（13.0）	
AML	16（39.0）	13（56.5）	
CMML	3（7.3）	2（8.7）	
MDS	8（19.5）	3（13.0）	
CAEBV	1（2.4）	0（0.0）	
PMF	1（2.4）	1（4.3）	
SAA	1（2.4）	1（4.3）	
移植情况［例（％）］			0.297
未移植	14（34.1）	5（21.7）	
移植后	27（65.9）	18（78.3）	
移植类型［例（％）］			1.000
同胞全相合	4（14.8）	2（11.1）	
单倍型相合	23（85.2）	16（88.9）	
移植后阶段［例（％）］			0.023
移植后晚期	15（55.6）	16（88.9）	
移植后早期	12（44.4）	2（11.1）	

**注** tNGS：靶向二代测序；CARV：社区获得性呼吸道病毒；ALL：急性淋巴细胞白血病；AML：急性髓系白血病；CMML：慢性粒-单核细胞白血病；MDS：骨髓增生异常综合征；CAEBV：慢性活动性EB病毒感染；PMF：原发性骨髓纤维化；SAA：重型再生障碍性贫血；移植后晚期：发病时间>移植后100 d；移植后早期：发病时间≤移植后100 d

二、病原分布

20例（31.25％）患者PCR CARV阳性，共检出7种24株病原，包括：鼻病毒4株、腺病毒2株、呼吸道合胞病毒7株、乙型流感病毒3株、偏肺病毒3株、甲型流感病毒2株、2019-nCoV 3株。

23例（35.94％）患者咽拭子tNGS检出CARV，共8种25株：鼻病毒6株、腺病毒2株、呼吸道合胞病毒5株、乙型流感病毒3株、偏肺病毒1株、甲型流感病毒2株、副流感病毒2株、2019-nCoV 4株。病毒分布及具体类型比较见图1。

**图1:**
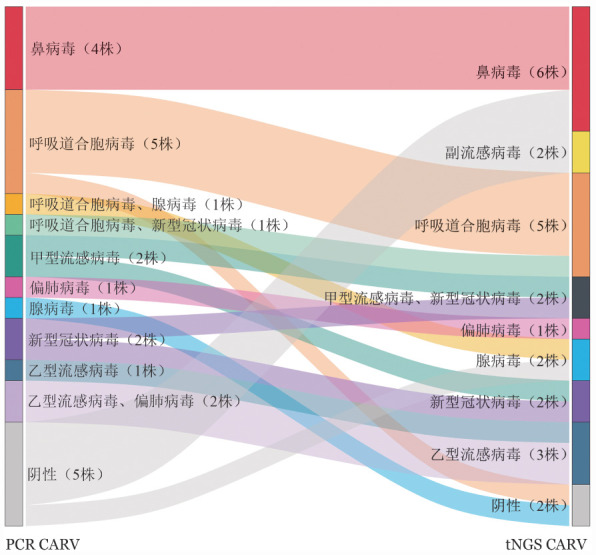
64例出现感染表现血液病患者中tNGS CARV及PCR CARV不一致检测结果比较 **注** tNGS：靶向二代测序；CARV：社区获得性呼吸道病毒

8例tNGS方法未检测出CARV病原，检出除CARV之外的病原包括：疱疹病毒5种36株：巨细胞病毒2株、人类疱疹病毒-7型4株、EB病毒20株、人类疱疹病毒-6型2株、单纯疱疹病毒-1型8株；肠道病毒1株；人类细小病毒B19 3株；细菌11种35株：具核梭杆菌10株、鲍曼不动杆菌10株、嗜麦芽窄食单胞菌1株、流感嗜血杆菌2株、咽峡炎链球菌群1株、肺炎克雷伯菌3株、阴沟肠杆菌复合群2株、铜绿假单胞菌3株、金黄色葡萄球菌1株、大肠埃希菌1株、非结核分枝杆菌1株；真菌3种12株：热带念珠菌2株、白念珠菌9株、近平滑念珠菌1株。

三、PCR与tNGS结果一致性比较

PCR CARV与tNGS CARV均阴性39例；PCR CARV与tNGS CARV均阳性17例，其中完全一致11例，部分一致6例：4例PCR检测出2种病原，tNGS检测到其中1种（未测出偏肺病毒2例、新型冠状病毒1例、呼吸道合胞病毒1例），2例tNGS检测出2种病原，PCR检测到其中1种（未测出甲型流感病毒1例、新型冠状病毒1例）；5例PCR CARV未检测到病原，tNGS CARV阳性（鼻病毒2例、副流感病毒2例、腺病毒1例），经临床医师判读考虑呼吸道CARV感染；3例PCR CARV阳性（腺病毒、呼吸道合胞病毒、甲型流感病毒各1例），2例tNGS阴性，1例tNGS检测到不同CARV（新型冠状病毒）。

以PCR为准，双阴性病例39例，双阳性病例17例，PCR阳性、tNGS阴性或检出病原不一致3例，PCR阴性、tNGS阳性5例，灵敏度为85.0％，特异度为88.6％，阳性预测值为77.3％，阴性预测值为92.9％，准确率87.5％，两种方法检测结果一致率较高（Kappa＝0.717，*P*<0.001）。

44例PCR CARV阴性样本中，tNGS检测出5例阳性（鼻病毒2例、副流感病毒2例、腺病毒1例），经临床医师判读考虑呼吸道CARV感染。20例PCR CARV阳性样本中，对应tNGS结果包括：11例PCR及tNGS检测出同一种CARV阳性；4例PCR检测2种CARV阳性，tNGS仅检测出其中1种（未测出偏肺病毒2例、2019-nCoV 1例、呼吸道合胞病毒1例）；2例tNGS阴性（未检测出腺病毒、呼吸道合胞病毒）；2例PCR检测1种CARV阳性，tNGS检测出另1种CARV阳性；1例PCR与tNGS检测结果不一致。

四、tNGS检测的临床意义

中位随访215.5（7，271）d，临床考虑呼吸道感染患者29例，2例患者死亡，1例死于原发病复发，1例死于呼吸道感染，最终肺部影像学无改善。其他原因引起发热/呼吸道改变患者35例，5例患者死亡，4例死于原发病复发，1例死于脑出血，5例中2例最终肺部影像学无改善。

29例诊断为呼吸道感染患者中，23例通过tNGS检测出CARV，余6例中2例应用PCR方法检测CARV阳性，1例诊断巨细胞病毒肺炎，2例病程中曾应用PCR方法查CARV阳性，1例患者临床考虑呼吸道感染；URTID 13例，LRTID 16例。

比较咽拭子tNGS CARV阳性及阴性患者，tNGS CARV阳性病例发热比例低于tNGS CARV阴性病例（*P*＝0.006），CARV阳性病例送检时HGB较阴性病例高（*P*＝0.033），WBC有高于阴性比例的趋势（*P*＝0.095），CRP、中性粒细胞绝对计数及PLT差异无统计学意义（表2）。

**表2:** 64例出现感染表现的血液病患者中tNGS CARV阴性与阳性病例感染相关特征比较

特征	tNGS CARV阴性（41例）	tNGS CARV阳性（23例）	*P*值
呼吸道感染［例（％）］	6（14.6）	23（100.0）	<0.001
发热［例（％）］	38（92.7）	17（73.9）	0.006
低氧［例（％）］	10（24.4）	7（30.4）	0.599
影像学改变［例（％）］	14（34.1）	10（43.5）	0.459
最差氧合指数［*M*（范围）］	318.5（88~458）	295（174~390）	0.333
最高CRP［mg/L，*M*（范围）］	60.3（0~200.0）	51.2（0~188.6）	0.334
WBC［×10^9^/L，*M*（范围）］	2.09（0.02~30.57）	4.02（0.03~19.18）	0.095
ANC［×10^9^/L，*M*（范围）］	1.19（0~6.24）	1.64（0~8.34）	0.237
HGB［g/L，*M*（范围）］	75（40~118）	81（58~148）	0.033
PLT［×10^9^/L，*M*（范围）］	51（7~230）	55（7~231）	0.314
存活［例（％）］	35（85.4）	22（95.7）	0.406

**注** tNGS：靶向二代测序；CARV：社区获得性呼吸道病毒；CRP：C反应蛋白

## 讨论

PCR是CARV感染诊断的标准方法，在发热的免疫抑制患者中应用广泛，tNGS作为一种高通量测序方法，比传统PCR具有更高的灵敏度和准确性[24]。既往比较tNGS与CMT结果的研究多在无菌体液和组织中展开[18]–[20],[25]，我们的研究结果提示，非侵入性采样的咽拭子样本使用tNGS检测CARV与PCR的结果一致性较好，对咽拭子样本进行tNGS检测可能为血液病患者提供一种快速且有用的诊断呼吸道CARV感染的方法。

本研究纳入64例具有感染表现的血液病患者，20例通过PCR方法检测出CARV阳性，呼吸道合胞病毒最常见（7/20），23例通过tNGS方法检测出CARV阳性，鼻病毒最常见（6/21），呼吸道合胞病毒其次（5/21）。与既往研究情况相似[2],[26]。以PCR结果为标准，评估tNGS敏感度为85.0％，特异度为88.6％，阳性预测值为77.3％，阴性预测值为92.9％，准确率87.5％，检测一致性好（Kappa＝0.717，*P*<0.001），提示tNGS对以病毒为主的常见呼吸道病原的诊断能力可能与PCR一致。

我们的研究选择了咽拭子作为评估的样本，具有快捷方便、易获得的特点，在健康人群开展的社区获得性肺炎病因研究，使用包括口/鼻咽拭子在内的不同类型的上呼吸道和下呼吸道样本，结果随样本类型的不同存在波动，因此我们的研究可能存在的问题是：对于LRTI，上呼吸道样本不一定代表下呼吸道致病病原，对于URTI，需要进一步在健康人群或无症状人群中测量病毒感染的背景患病率才能阐明检测结果是否有意义。此外还有研究认为，鼻咽拭子的敏感性高于咽喉拭子[27]–[30]；Pérez等[2]分析了353起CARV-LRTI事件，并将其分为可能的LRTID和已证实的LRTID，在199例通过上呼吸道标本诊断的拟诊CARV-LRTID中，40例进行了肺泡灌洗，结果均为阴性，但死亡率与通过肺泡灌洗液确诊的CARV-LRTID类似。Rachow等[31]进行的前瞻性研究采集造血干细胞移植患者和健康对照组口咽含漱液应用PCR方法进行CARV检测，移植患者CARV阳性率明显高于健康人群，出现URTI表现与CARV阳性相关；因此咽拭子尽管存在不可避免的局限性，但对其进行CARV检测，对病因研究有一定参考价值。

我们观察到纳入的患者中，CARV感染患者血细胞较非感染患者偏高，分析其原因包括：移植患者中，CARV感染多发生在移植后晚期，与移植后免疫重建趋势一致，同时，纳入的非移植患者，多处于粒细胞缺乏或原发病引起血象下降阶段。tNGS能够覆盖除呼吸道病毒以外的多种病原，包括可以引起肺部病变的人类疱疹病毒6型、巨细胞病毒等，以及致病性并不确定的细小病毒等[1]。在移植患者中，有研究发现肺泡灌洗液存在致病性不确定的病原，死亡率与存在致病性确定的呼吸道病原类似[32]，咽拭子tNGS检测到这些病原的意义，值得在今后的研究中继续探索。

本研究局限性包括：①只比较了咽拭子部分病毒PCR的结果，咽拭子采样过程造成的假阴性及假阳性均较无菌部位标本多，在细菌、真菌感染以及对于可能致病的非呼吸道病毒如疱疹病毒的诊断价值有限；②缺乏客观的感染判定标准，阻碍了tNGS灵敏度和特异度的计算，影响对其诊断性能的全面评估；③tNGS技术缺乏统一的病原学诊断标准，对阳性结果判读、区分微生物定植和感染需要更多研究；④纳入患者情况多样，样本量相对较小，患者随访时间短，研究时长有限。
